# Examining the Relationship between Dietary Intake, Socioeconomic Status, and Systolic Blood Pressure of Adults on Hemodialysis in Quito, Ecuador

**DOI:** 10.1016/j.cdnut.2023.102047

**Published:** 2023-11-24

**Authors:** Lucia Eguiguren-Jiménez, Sofia Acevedo, Jeanette M Andrade

**Affiliations:** Food Science and Human Nutrition Department, University of Florida, Gainesville, FL, United States

**Keywords:** hemodialysis, Ecuador, dietary intake, socioeconomic status, blood pressure

## Abstract

**Background:**

In Ecuador, the number of adults on hemodialysis (HD) continues to rise. Currently, the effect dietary habits and socioeconomic status (SES) have on blood pressure is not known for those on HD.

**Objectives:**

The objectives of this study focused on adults on HD in Quito, Ecuador to *1*) assess the relationship between dietary intake and SES; *2*) compare dietary intake to the Kidney Disease Outcomes Quality Initiative (KDOQI) guidelines; and *3*) explore the relationship between dietary intake and systolic blood pressure.

**Methods:**

This cross-sectional study was conducted at the dialysis center within Eugenio Espejo Specialties Hospital in Quito, Ecuador between May and June 2022 among 50 adults on HD. Three 24-h recalls were used to determine average dietary intake and the 25-item Instituto Nacional de Estadística y Censos-Stratification of SES was used to determine SES. Electrolytes and 2 blood pressure readings were collected. Qualitative narrative data analysis was performed to identify themes using NVivo v12. T-tests of independence, simple, and multiple linear regressions using age and sex as confounders were conducted using R and a *P <* 0.05 was deemed as statistically significant.

**Results:**

On an average, 76% of participants consumed <25 kcal/kg body weight energy and 64% consumed <1 g protein/kg body weight. Participants consumed less energy and protein compared with the KDOQI guidelines (*P <* 0.05). Positive relationships were observed with potassium and blood pressure (β = 0.020, *P <* 0.05) and SES with energy, protein, and phosphorus (*P <* 0.05). Themes that were identified as contributing to dietary intake were limited knowledge, lack of consistency with dietary information, and limited appetite.

**Conclusions:**

Findings from this study indicate that the focus should be on improving energy and protein intake for this population.

## Introduction

Globally, hemodialysis (HD) is the most common treatment for individuals who are at the final stage of kidney disease [1]. Moreover, the prevalence of adults on HD continues to rise because in 2010, an estimated 2 million were on HD with the projected rise to be more than double by 2030 [2]. Several factors are contributing to this rise with one being access to dialysis in low-resourced countries [3] such as Ecuador. The Pan American Health Organization and the WHO report that in Ecuador, deaths related to kidney disease were 37.4 deaths per 100,000 people, which have deemed this being the fourth leading cause of death [4]. Furthermore, the Ecuadorian national guideline for chronic kidney disease (CKD) prevention, diagnosis, and treatment states that the local prevalence of individuals on dialysis is 1074 per million population [5].

Ecuador is divided into 4 main regions: Amazonia, Costa, Galapagos Islands, and Sierra, which can be further divided into 26 provinces (for example, Pichincha and Guayas). Quito, the capital of Ecuador, is in the region of Sierra within the Pichincha province. A recent study demonstrated that the prevalence of CKD among adults (*n =* 813), who resided in Quito and visited a clinic during the years 2019–2021, was 7.2% [6]. Through this same study, a subanalysis of 553 adults revealed that those with systolic blood pressure (SBP) ≥140 mm Hg and resided in rural areas had higher risk for CKD compared with those with SBP <140 mm Hg and resided in an urban area [6]. Furthermore, in 2016, deaths associated with CKD accounted for more than 200,000 people [7]. Deaths attributed to these noncommunicable diseases are likely because of lack of access to healthcare as 40.9% of Quito’s population is not insured [8]. Even though 48.8% have access to the general insurance (Instituto Ecuatoriano de Seguridad Social (IESS)) [7], many do not take advantage of seeking medical attention because of wait times and the inability to afford the medical expenses once diagnosed with a disease [8]. Beyond these factors, a poor diet quality (lack of fruits, vegetables, and consuming foods high in sodium) may contribute to CKD [[9], [10], [11], [12], [13], [14]], which is seen more often in adults classified as low socioeconomic status (SES) and who have limited educational opportunities [12,14].

International guidelines for CKD, such as Kidney Disease Improving Global Outcomes and Kidney Disease Outcomes Quality Initiative (KDOQI), emphasize consuming adequate energy (25–35 kcals/kg body weight daily) and protein (1.0–1.2 g/kg body weight daily) and micronutrients to improve outcomes when on HD [[15], [16], [17]]. However, most adults consume an average of 0.9 g protein/kg body weight daily [15], which may lead to protein energy malnutrition, inflammation, and further complications [15,18,19]. Consuming a balance of sodium and potassium is critical to reduce potential elevations in blood pressure [15,20]. Results from studies are inconsistent, though, with the relationship between consuming sodium and potassium on blood pressure [[21], [22], [23], [24]].

For studies that have focused on the relationship between energy and nutrient consumption and outcomes of adults on HD, results may be inconsistent because of the methods used to collect dietary information such as food frequency questionnaires, 24-h dietary recalls, or focused only urinary/serum levels [21]. The KDOQI guidelines suggest that a 3-d food record is the preferred method to assess diet for adults with CKD and it should be conducted with a trained professional for more accurate findings [15]. As opposed to resource-sufficient countries such as United States and Europe that have shown a relationship between SES, dietary habits, and blood pressure [[25], [26], [27], [28]], limited studies have been conducted in Ecuador. The objectives of this study focused on adults on HD in Quito, Ecuador, to *1*) assess the relationship between dietary intake (total energy, total protein, sodium, potassium, and phosphorus) and SES; *2*) compare dietary intake (total energy, total protein, sodium, potassium, and phosphorus) to the KDOQI guidelines; and *3*) explore the relationship between dietary intake (total energy, total protein, sodium, potassium, and phosphorus) and SBP. The overall hypothesis was that adults on HD who were classified as low SES would have higher SBP and lower adherence to the KDOQI guidelines compared with those who were classified as high SES.

## Methods

### Participants

A cross-sectional study was conducted at the dialysis center within Eugenio Espejo Specialties Hospital in Quito, Ecuador between May and June 2022. This hospital was used because it is considered a public hospital and accepts individuals with or without insurance [29], thus it would include participants with differing levels of SES. This study was approved by the Ethics and Research Committee of Human subjects of CEISH-USFQ (E01-EX066-2022-CEISH-USFQ) and by the University of Florida Institute Review Board (IRB202102501).

Recruitment of participants was initially identified through the head physician of the dialysis center. Inclusion criteria included adults who were *1*) 18 y of age or older, *2*) spoke Spanish fluently, *3*) had recent (within 1 mo) serum creatinine, sodium, and potassium, *4*) blood pressure readings upon arrival and initiation of dialysis, and *5*) were receiving HD treatments at least twice a week. Participants who did not meet the inclusion criteria and/or were considered pregnant or lactating were excluded from the study. After the initial contact with the physician, participants who showed interest in the study were screened by the investigator, and if qualified, signed a consent form. From the census of the dialysis center during the months of this study, a total of 60 potential participants were contacted, of whom 50 agreed to participate.

### Study procedures

Participants completed 3–24-h recalls and the Instituto Nacional de Estadística y Censos (INEC)-Stratification of SES.

### 24-h recalls

Three 24-h recalls were collected by the investigator who is a trained dietitian in Ecuador. The recalls were collected following the KDOQI guidelines [15]—2 collected on a dialysis day and 1 on a non-dialysis day. Each 24-h recall lasted an average of 20 min using the multiple-pass technique [30]. The multiple-pass technique consists of 3 rounds of questions and conversations about food and beverage intake. First, participants were asked to recall all the foods/beverages they consumed in the past 24 h, where they specified the time of consumption, such as breakfast, lunch, dinner, morning, or afternoon snacks. On the second pass, the investigator went back through the foods/beverages mentioned and asked about the quantity and how the foods were prepared (oil, salt, etc.). On the third pass, the investigator asked the participants if any other food/beverage was consumed, and mentioned some examples of commonly forgotten items, such as chocolate, soft drinks, snacks, or condiments, and added those items to the 24-h recall. Lastly, participants were asked to indicate where they consumed these meals and/or snacks. The reported food and beverage quantities from those 3-d were averaged and converted into kcals, g or mg of nutrients based on Elizabeth Stewart Hands and Associates’ Food Processor Software [31]. The main nutrients of interest for this study were energy, protein, sodium, potassium, and phosphorus. During the interview, the investigator (LE-J) asked open-ended questions about how participants felt while on HD regarding appetite/gastrointestinal health and in general life while on HD that was used for qualitative narrative data analysis. These questions were included as a routine standard of care that typically the medical director would ask of the HD population.

### INEC-Stratification of SES

The INEC-Stratification of SES was created and validated by INEC, an Ecuadorian organization that oversees all the statistics of the population [31]. This survey has a total of 25 items that includes 6 dimensions: possession of goods (7 items), housing characteristics (5 items), consumption habits (5 items), access to technology (4 items), economic activity (3 items), and education (1 item). Each dimension has a different scoring scale with a minimum score consistently being 0. For example, the housing characteristics have a maximum score of 236, whereas consumption habits have a maximum score of 99. Each dimension is then summed for an overall SES of a maximum of 1000, which ranges from a grade of A (845.1–1000 points) to a D (0–316 points) with the highest grade indicating higher SES and the lowest grade indicating lower SES [32].

### Statistical analysis

Demographics of the participants, such as age, sex, education level, and insurance, were presented as frequencies and characterized with descriptive analyses. Each participant’s SES was evaluated and classified into low (level D of INEC-SES), middle (levels C- and C) or high (level B and A) SES, which followed the criteria of a similar study [33]. This allowed the investigator to compare results with international classification of SES. For dietary intake, adequacy was established by comparing mean values of energy, protein, sodium, potassium, and phosphorus, to the KDOQI dietary recommendation. For energy and protein, estimations were conducted based on participants’ heights and weights, which were provided in the medical chart. T-tests with paired data were used to compare dietary habits with the KDOQI guidelines.

Simple linear regressions (Y= a + bX) were used to examine the relationships between SES and dietary intake, where “Y” represented each dependent variable as a different nutrient (for example, protein, potassium) and “X” represented SES. Also, simple linear regressions were used to explore the relationships between dietary intake and systolic BP. The “Y” represented systolic BP and “X” represented the different nutrients. Normality was assessed for the regression by performing Shapiro-Wilk test. Furthermore, multiple linear regression models were used to explore the relationships between dietary intake and systolic BP based on a different predictor. The first model, Y= a + b1X1 + b2X2 + b3X3, analyzed systolic BP as the dependent variable “Y”, and X1, X2, and X3, which represented sodium, potassium, and phosphorus, respectively. The second model analyzed the same variables, but age (X4) and sex (X5) were included to assess possible confounders. Statistical significance was determined at *P <* 0.05 using R version 4.1.2 [34].

Open-ended responses from the participants regarding their dietary habits and life while on HD were analyzed using a qualitative narrative data analysis approach [35] using NVivo v12 [36]. Transcripts of information from the participants were transcribed into NVivo 12 before the analysis. The qualitative narrative data analysis approach was completed in 3 phases. The first phase involved the preliminary analysis, in which case the investigators (LE-J and JMA) read through the transcripts to identify stories/topics, summarized these stories/topics and then constructed each individual story/topic into themes using the Narrative Process Coding System [37]. The second phase involved a micro-analysis of the themes from the 3 investigators (LE-J, JMA, and SA) to identify the subcontext of each transcript based on cultural narratives, voices from different ages and genders, and text structure analysis. Discrepancies about the identification of the themes or microanalyses were sorted through multiple discussions among the reviewers until agreements were reached [38]. The third phase involved the investigator (LE-J) summarizing and interpreting the content within the themes.

## Results

### Sample characteristics

A majority of participants were males (60%) with a mean age (±SD) of 52.24 (±16) y, and 66% were classified as middle SES (316.1–696), 28% of participants completed high school, and none of the participants had insurance (Table 1). Mean serum values of sodium and potassium were considered within normal range of 134.7 ± 5.81 mEq/L (135–145 mEq/L normal) and 4.34 ± 0.77 mEq/L (3.7–5.2 mEq/L), respectively.

**TABLE 1:** Demographic characteristics

Group	*N* =50	Percentage
Sex		
Males	30	60
Females	20	40
Age (y)		
18–44	18	36
45–59	15	30
>60	17	34
Education level		
No education	3	6
Incomplete elementary school	10	20
Complete elementary school	10	20
Incomplete high school	7	14
Complete high school	14	28
3 y or less of undergraduate studies	3	6
4 or more year of undergraduate studies	3	6
Graduate degree	0	0
Marital status		
Single	18	36
Married	18	36
Divorced	7	14
Co-habitant	2	4
Widowed	1	2
Did not specify	4	8
Insurance		
Uninsured	50	100
Private or public insurance	0	0

### Dietary intake

Participants, on average, consumed 1172.72 ± 449.33 kcal, 50.86 ± 18.15 g of protein, 2048.34 ± 997.98 mg sodium, 1117.16 ± 531.75 mg potassium, and 613.95 ± 211.29 mg of phosphorus. For energy requirements, 38 participants (76%) consumed less than 25 kcals/kg body weight and for protein, 32 participants (64%) consumed less than 1 g/kg body weight. As for sodium, potassium, and phosphorus, when compared with the KDOQI recommendations, the majority of the population (83%) were under the limits of these values (Table 2). Furthermore, t-tests for paired data showed that participants consumed less total energy [t(49) = −3.54, *P <* 0.001] and total protein [t(49) = −2.51, *P <* 0.05], compared with the KDOQI guidelines. A t-test for independence showed that the participants classified as low SES consumed less total energy compared with participants classified as middle SES t(29.91) = −2.35, *P <* 0.05, less total protein t(34.38) = −2.91, *P <* 0.01 and phosphorus t(30.68) = −2.72, *P <* 0.05.

**TABLE 2:** Energy and nutrients intake compared with KDOQI guidelines (*n* = 50)

Variables	Mean dietary values (±SD)	KDOQI recommendations	Adequate *N* (%)	Inadequate *N* (%)
Energy (Kcal/d)	1172.7 (±449.3)	—		
Energy (Kcal/kg/d)	21.3 (±10.6)	25–35	12 (24)	38 (76)
Protein (g/kg/d)	0.9 (±0.4)	1.0–1.2	18 (36)	32 (64)
Sodium (mg/d)	2048.3 (±997.9)	<2300	36 (72)	14 (28)
Potassium (mg/d)	1117.2 (±531.8)	<3000	50 (100)	0 (0)
Phosphorus (mg/d)	613.9 (±211.3)	<800	39 (78)	11 (22)

Abbreviation: KDOQI, Kidney Disease Outcomes Quality Initiative.

Simple linear regression analyses showed that SES was positively related with total energy (β = 313.10, *P <* 0.05), total protein (β = 14.57, *P <* 0.01), and phosphorus (β = 165.99, *P <* 0.01) (Table 3). There were positive relationships with SBP and potassium (β = 2.10e^−02^, *P <* 0.01) and phosphorus (β = 0.04, *P <* 0.05) (Table 4). The covariates sex and age did not influence these associations.

**TABLE 3:** Nutrients associated with SES (*n* = 50)

Coefficient	Estimate (95% CI)	SE	*t* value	Pr (>|t|)
Average calories^1^				
(Intercept)	653.01 (204.42, 1101.56)	223.10	2.93	<0.01∗∗
SES	313.10 (53.24, 572.94)	129.20	2.42	0.02∗
Average protein^2^				
(Intercept)	26.67 (8.91, 44.43)	8.83	3.02	<0.01∗∗
SES	14.57 (4.29, 24.86)	5.12	2.85	<0.01∗∗
Average phosphorus^3^				
(Intercept)	338.41 (131.01, 545.82)	103.15	3.28	<0.01∗∗
SES	165.99 (45.84, 286.13)	59.76	2.78	<0.01∗∗

Abbreviations: CI, confidence interval; SES, socioeconomic status.

∗∗*P* value < 0.01.

∗*P* value < 0.05.

1 Simple linear regression: R^2^ = 0.108, R^2^ adjusted = 0.090, *P <* 0.05.

2 Simple linear regression: R^2^ = 0.144, R^2^ adjusted = 0.126, *P <* 0.01.

3 Simple linear regression: R^2^ = 0.138, R^2^ adjusted = 0.120, *P <* 0.01.

**TABLE 4:** Nutrients associated with systolic blood pressure (*n* = 50)

Coefficient	Estimate (95% CI)	SE	*t* value	Pr (>|t|)
Average sodium^1^				
(Intercept)	129.30 (111.56, 147.12)	8.84	14.63	<0.001∗∗∗
Sodium	5.14e^−03^ (−0.00, 0.01)	3.88 e^−03^	1.33	0.19
Average potassium^2^				
(Intercept)	116.40 (99.60, 133.27)	8.38	13.90	<0.001∗∗∗
Potassium	0.02 (0.01, 0.04)	6.77e^−03^	3.10	<0.01∗∗
Average phosphorus^3^				
(Intercept)	117.47 (94.10, 140.84)	11.63	10.11	<0.001∗∗∗
Phosphorus	0.04 (5.06e^−04^, 0.07)	0.02	2.04	<0.05∗

∗∗∗*P* value < 0.001.

∗∗*P* value < 0.01.

∗*P* value < 0.05.

1 Simple linear regression: R^2^ =0.035, R^2^ adjusted = 0.015, *P* > 0.05.

2 Simple linear regression: R^2^ =0.167, R^2^ adjusted = 0.150, *P <* 0.01.

3 Simple linear regression: R^2^ =0.080, R^2^ adjusted = 0.061, *P <* 0.05.

An unadjusted multiple linear regression model showed that only potassium (β = 0.02, *P <* 0.05) remained significant with SBP (Table 5). An adjusted multiple linear regression model that controlled for age and sex, did not change the association between SBP and potassium (β = 0.02, *P <* 0.05), and there remained a non-statistical difference with the other nutrients (Table 5).

**TABLE 5:** Nutrients associated with systolic blood pressure

	Estimate (95% CI)	SE	*t* value	Pr (>|t|)
Model 1: coefficient				
(Intercept)	115.10 (92.26, 137.93)	11.35	10.14	<0.001∗∗∗
Sodium	−0.00 (−0.012, 0.01)	0.01	−0.46	0.65
Potassium	0.02 (0.00, 0.04)	0.01	2.26	<0.05∗
Phosphorus	0.01 (−0.04, 0.06)	0.03	0.40	0.69
Model 2: coefficient				
(Intercept)	131.88 (93.65, 170.10)	18.97	6.95	<0.001∗∗∗
Sodium	−0.00 (−0.01, 6.46e^−03^)	0.01	−0.74	0.46
Potassium	0.02 (0.00, 0.04)	0.01	2.19	<0.05∗
Phosphorus	0.02 (−0.04, 0.07)	0.03	0.65	0.52
Age	−0.23 (−0.71, 0.25)	0.24	−0.96	0.34
Sex	−8.66 (−24.30, 6.99)	7.76	−1.12	0.27

Multiple linear regression (Model 1): R^2^ =0.172, R^2^ adjusted = 0.118, *P <* 0.05; multiple linear regression (Model 2): R^2^ =0.209, R^2^ adjusted = 0.120, *P =* 0.05.

∗∗∗*P* value < 0.001.

∗*P* value < 0.05.

### Qualitative narrative results

Analysis of the qualitative feedback indicated 3 main themes toward dietary intake—knowledge, communication, and adverse events. All participants indicated that they received no formal nutritional counseling and, thus, were unsure of the types of foods to consume while on HD. Adding to this uncertainty, participants expressed confusion because different medical providers informed them to avoid certain foods such as fruit juice and red meat, yet to make sure they ate fruit and more protein. Moreover, the participants addressed that after HD, symptoms such as nausea and dizziness provoked a loss of appetite. For this reason, many participants indicated that they consumed 1–2 meals a day, did not consume foods/beverages at dialysis or post-dialysis for a few hours, and rarely snacked in between meals. Furthermore, another theme arose about life while on HD which was traveling. Seven participants commented that it was challenging to attend HD because of their travel arrangements, which in some cases took more than 3 h.

## Discussion

This cross-sectional study aimed to assess the relationship between SES and dietary intake, compare dietary intake with the KDOQI guidelines, and explore the relationship between dietary intakes on SBP of adults on HD in Quito, Ecuador. Results illustrated that participants consumed less total energy and total protein compared with the recommendations from the KDOQI guidelines. Furthermore, participants classified as low SES consumed less energy and total protein, potassium, and phosphorus compared with those classified as middle SES. Positive relationships were observed with SES and total energy, total protein, and phosphorus, and with SBP and potassium. Neither sex nor age had an influence on these relationships.

Diet may reduce the progression of and improve outcomes associated with kidney disease. The results from this study showed that participants had an average total energy intake of 21.3 kcal/kg/d and an average total protein intake of 0.9 g/kg/d that was less than the recommendations of 25–35 kcal/kg body weight for total energy and 1.0–1.2 g/kg body weight for total protein [15]. Similar to results from this study, the 2012 Ecuadorian National Health and Nutrition Survey (ENSANUT) showed that within the Sierra region, where Quito is located, average total energy intake was 1729.5 kcals and average total protein intake was 54g, which was lower than other Ecuadorian regions [39]. The authors suggested that individuals who reside in the Sierra region may have less access, resources, and economic opportunities compared with individuals who reside in other regions of Ecuador [39]. A cross-sectional study was performed in Italy with adults on HD (*n =* 37) to determine a relationship between dietary intake, using 3-d diet diaries, to age, weight, and others. Results showed that regardless of age and weight, energy intake was, on average, 24.9 ± 10.1 kcal/kg and 0.64 ± 0.4 g of protein/kg, which were lower than this study’s results [40]. A cross-sectional study was conducted in Seoul, South Korea among adults on HD (*n =* 63) to determine dietary intake using 3-d food records. Results showed that participants consumed an average energy intake of 21.9 kcal/kg/d and protein intake of 0.9 g/kg/d, which were closer to the results of this study. The authors provided several reasons for their findings such as a monotonous diet of limited diversity because of the restrictions, limited appetite, and potential adherence issues related to not knowing what to eat [41]. Similarly, in this study, participants mentioned the a of appetite, especially after HD treatment, and a lack of knowledge of what to eat. Moreover, most participants expressed consuming only 2 meals when they had HD treatment because of the schedule of the treatment that interfered with their daily routine. Further research needs to be focused on strategies to enhance total energy and total protein intake among the HD population.

In addition to total energy and total protein, sodium, potassium, and phosphorus are also important nutrients for the management of this disease. Results from this study showed that participants consumed the recommendations for sodium, potassium, and phosphorus. Even though most participants met the requirements, controversy exists on whether underconsuming these nutrients could worsen nutritional status and increase mortality risk while on HD. For instance, potassium has been a micronutrient of discussion among CKD researchers for its benefits in reducing blood pressure, specifically SBP, and overall cardiovascular health [42]. During end-stage kidney disease, potassium is generally restricted because of the kidney’s incapability to adequately filter, thus accumulating within the body, causing several complications such as arrythmias, muscle weakness, cardiac arrest, among others [43]. To the contrary, it has been observed that the underconsumption of this electrolyte has negative impacts on adults with chronic diseases, including CKD [44,45]. For example, a prospective multicenter study that included adults on HD (*n =* 415) showed that those with lower dietary intake of potassium (543 ± 221 mg) had a 2.5-fold higher risk of mortality compared with those consuming higher amounts, and were independent of other comorbidities, sociodemographic characteristics, and dialysate potassium concentrations [45]. Furthermore, a critical review of the different recommendations and restrictions made about the key nutrients in CKD acknowledged that either hypo- or hyperkalemia could be detrimental for adults on HD, both increasing risk for mortality [46]. In this study, participants had a low dietary intake of potassium, because the consumption of fruits and vegetables was almost null. This can be attributed to the lack of nutritional counseling and the dietary restrictions generally prescribed by the medical provider as mentioned by the participants.

Results showed that potassium intake was positively associated with SBP, independently from dietary intake of sodium and phosphorus, age, and sex. This differs from other studies that revealed that potassium is inversely related to blood pressure, thus, a higher consumption will result in lower blood pressure [[47], [48], [49]]. The exact mechanism by which potassium influences blood pressure remains unknown. One theory is the interaction between sodium and potassium within the membrane influences the sodium-chloride cotransporter [50]. However, this mechanism is explained under normal physiological conditions where the body can maintain homeostasis and may be a different mechanism when one has CKD. The main findings from this study demonstrated that the participants were compliant with the KDOQI recommendations, and consumed on average, half of what is recommended without abnormal serum potassium, yet the relationship between dietary potassium intake and SBP remained positive. Possible explanations for these results include the combination of lower sodium and potassium intake that could diminish the effect of potassium as a lowering blood pressure agent [45,48], differentiation in determining SBP such as frequency and measurement techniques [51], medication [52], dialysis adequacy parameters, and/or dialysate of potassium administered to each individual [53]. Because limited clinical trials have focused on the relationship between dietary potassium and blood pressure among this population, it is an area to pursue to identify the mechanism of action to provide more tailored guidelines for adults on HD.

Likewise, participants consumed, on average, 186.05 mg of phosphorus that met the recommendations of <800 mg of phosphorus daily. This low dietary intake was attributed to participants restricting their consumption of meat, dairy, vegetables, and some legumes because of recommendations from medical professionals. In CKD, dietary phosphorus is restricted because of possible accumulation of this nutrient in the body. Phosphorus bioavailability is higher in processed foods compared with organic sources such as animal or plant-based food items [54,55], which may contribute to elevated serum phosphorus that would then stimulate the production of growth factor-23 and the parathyroid hormone and, thus, contributing to overall loss of bone mineral density [56]. In this study, participants consumed limited organic and inorganic sources of phosphorus. These results are in accordance with ENSANUT survey, where males and females from different age groups did not meet international dietary phosphorus recommendations, regardless of the source. Moreover, the ENSANUT results highlighted that the consumption of dairy progressively diminished as aged progressed [39], which was also present in this study. This may have been due to the age of the participants and the foods they were informed to avoid while on HD. Nonetheless, restricting phosphorus intake to lower serum phosphorus levels and prevent negative consequences are still debated. A retrospective study that analyzed 30,075 records of adults on HD throughout 507 outpatient HD centers demonstrated that the reduction of serum phosphorus by reduced intake of dietary phosphorus increased risk of mortality and reduced survival rates [57]. Furthermore, the main findings from the IMPROVE-CKD Australian study with 90 participants showed that despite high levels (>1471 mg) of total dietary phosphorus intake, no effect was observed on biomarkers of CKD-mineral and bone disorder [58]. Overall, dietary restrictions must be carefully considered, because it could compromise the nutritional status of adults on HD. As serum phosphorus was not a routine biochemical marker within this HD center, the impact of dietary phosphorus intake on serum levels is not known; thus, recommendations to collect this biomarker for tailored dietary recommendations must be considered.

This study demonstrated that SES was positively related to total energy, total protein, and phosphorus intake. Participants classified as low SES tended to consume less total energy and nutrients, whereas those with middle SES consumed higher total energy and nutrients. In agreement with these results, a cross-sectional study using NHANES data from 1999–2002 examined the effect SES had on several factors including dietary intake of elders (*n =* 2675). Results demonstrated that the elders who were classified in the low and middle SES group (*n =* 1962) consumed at least 125 calories less than elders in the higher SES group (*n =* 713). Furthermore, elders in the low SES group consumed less protein, but solely because of the inability to buy or obtain an adequate amount of food, rather than the consumption of protein being inadequate by itself [59]. Moreover, the Healthy Aging in Neighborhoods of Diversity across the Life Span study of 2058 adults examined the associations between Dietary Approaches to Stopping Hypertension (DASH) diet adherence and living or not in poverty to CKD. Results revealed that the participants who were living in poverty were less adherent to the DASH diet and were considered at a high risk for CKD compared with those not living in poverty. This indicated that people living in poverty were unable to obtain the foods needed to minimize CKD [60]. Although not an HD population, another cross-sectional study that used data from NHANES III (*n =* 14,261) examined the impact of SES on dietary phosphorus intake. Results demonstrated that individuals who were considered in the low SES quartiles had lower dietary phosphorus intake compared with those in higher SES quartiles [54]. In this study, it is not known if the dietary phosphorus impacted serum phosphorus because no serum phosphorus levels were routinely collected at the HD clinic.

In this study, participants classified as low SES mentioned that they did not have funds to purchase foods, had low food availability near their place of residence, and had limited appetite and thus consumed smaller portion sizes than required for dietary needs. Moreover, SES is known to be a determinant of education level, household characteristics, residence area, among others [61]. These components are associated with health outcomes, especially those diagnosed with chronic diseases, like CKD. Several studies [26,[62], [63], [64]] demonstrated that limited education, income restrictions, behavioral factors, and residence location (rural vs. urban), influenced the progression and management of a disease. These studies concluded that education was strongly associated with reduction and prevention of complications associated with chronic diseases. In this study, most participants had an education level of high school or lower (82%), and only 12% reached college, which could explain why many were unaware of the foods/beverages they needed to consume. A study that analyzed 61,457 records from participants enrolled in the Kidney Early Evaluation Program demonstrated that education level was highly associated with reduction of kidney function and mortality [65]. A systematic review and meta-analysis of 14 studies confirmed that education was significantly associated with mortality among countries that had limited healthcare resources [66]. Furthermore, education is a determinant of diet intake [67], in which lower educational attainment may contribute to the progression of chronic diseases. From the qualitative feedback, 10 participants were not aware of the reason for being on dialysis and most participants were unsure about foods to consume while on dialysis. In congruence with the results, a multicentric study performed in Slovakia within 20 dialysis centers (*n =* 452), showed that low health literacy was significantly associated with poor adherence to dietary recommendations, fluid intake, among others. The main findings showed that not “having sufficient information to manage health” was associated with higher levels of serum phosphate. Also, participants with less “ability to actively manage their health” and less “ability to actively engage with health care providers” reported nonadherence to dietary recommendations and were overhydrated [68]. A semistructured, interview that included 4 remote indigenous communities within Canada aimed to explore food-access barriers of people diagnosed with CKD stage 5 and end-stage kidney disease (*n =* 36). The main findings demonstrated that lack of education/information was a barrier that participants encountered to follow dietary recommendations for these stages [14], which is similar to the results from this study. Further nutrition education interventions should focus on the impact knowledge has on dietary adherence and progression of disease state for those individuals with limited educational attainment.

Overall, this study presents some limitations. First, limited routine serum markers are normally performed of adults on HD in Quito, which inhibited the ability to expand the findings from dietary intake to serum levels. Thus, policies and practices need to be in place to ensure that routine lab work is performed to minimize risk of CKD and the progression of this disease. Second, the software used for the dietary recalls did not provide information for specific native food items from Ecuador, thus, other similar food items were selected, which might have influenced the results of dietary intake. Third, results cannot be comparable with other studies performed in Ecuador or Ecuador’s populated cities that can further contribute to elucidating the associations found in this study because of the population included within this study.

In conclusion, this study is one of the few studies that focused on SES, dietary habits, and blood pressure of adults on HD. Even though the results of this study only focused on one of the populated cities in Ecuador, this study could serve as a foundation for future studies to be performed in other cities of Ecuador as well as high-quality population-based studies that elucidate CKD’s situation around the country. Overall, the main findings from this study suggest that future research should focus on the impact of well-established nutrition education programs that prevent CKD and its progression to dialysis treatments, which could avoid the great economic and physical burden individuals suffer from when undergoing dialysis treatment and further developing other complications that adds to CKD health outcomes.

## Author contributions

The authors’ responsibilities were as follows – LE-J, JMA: conceived the study design; LE-J: collected data, completed data and statistical analysis, and initially wrote the manuscript; and all authors: reviewed and commented on subsequent drafts of the manuscript and read and approved the final manuscript.

## Funding

This work was supported by USDA National Institute of Food and Agriculture Hatch, FLA-FOS-00599 and by the Tinker Foundation for awarding Field Research Grant.

## Data availability

Data described in the manuscript, code book, and analytic code will be made available upon request pending application and approval.

## Conflict of interest

The authors report no conflicts of interest.
